# Accuracy of Diabetic Retinopathy Staging with a Deep Convolutional Neural Network Using Ultra-Wide-Field Fundus Ophthalmoscopy and Optical Coherence Tomography Angiography

**DOI:** 10.1155/2021/6651175

**Published:** 2021-04-03

**Authors:** Toshihiko Nagasawa, Hitoshi Tabuchi, Hiroki Masumoto, Shoji Morita, Masanori Niki, Zaigen Ohara, Yuki Yoshizumi, Yoshinori Mitamura

**Affiliations:** ^1^Department of Ophthalmology, Saneikai Tsukazaki Hospital, Himeji 671-1227, Japan; ^2^Department of Technology and Design Thinking for Medicine, Hiroshima University, Hiroshima 739-8511, Japan; ^3^Graduate School of Engineering, University of Hyogo, Kobe 657-0013, Japan; ^4^Department of Ophthalmology, Institute of Biomedical Sciences, Tokushima University Graduate School, Tokushima 770-8851, Japan

## Abstract

**Purpose:**

The present study aimed to compare the accuracy of diabetic retinopathy (DR) staging with a deep convolutional neural network (DCNN) using two different types of fundus cameras and composite images.

**Method:**

The study included 491 ultra-wide-field fundus ophthalmoscopy and optical coherence tomography angiography (OCTA) images that passed an image-quality review and were graded as no apparent DR (NDR; 169 images), mild nonproliferative DR (NPDR; 76 images), moderate NPDR (54 images), severe NPDR (90 images), and proliferative DR (PDR; 102 images) by three retinal experts by the International Clinical Diabetic Retinopathy Severity Scale. The findings of tests 1 and 2 to identify no apparent diabetic retinopathy (NDR) and PDR, respectively, were then assessed. For each verification, Optos, OCTA, and Optos OCTA imaging scans with DCNN were performed.

**Result:**

The Optos, OCTA, and Optos OCTA imaging test results for comparison between NDR and DR showed mean areas under the curve (AUC) of 0.79, 0.883, and 0.847; sensitivity rates of 80.9%, 83.9%, and 78.6%; and specificity rates of 55%, 71.6%, and 69.8%, respectively. Meanwhile, the Optos, OCTA, and Optos OCTA imaging test results for comparison between NDR and PDR showed mean AUC of 0.981, 0.928, and 0.964; sensitivity rates of 90.2%, 74.5%, and 80.4%; and specificity rates of 97%, 97%, and 96.4%, respectively.

**Conclusion:**

The combination of Optos and OCTA imaging with DCNN could detect DR at desirable levels of accuracy and may be useful in clinical practice and retinal screening. Although the combination of multiple imaging techniques might overcome their individual weaknesses and provide comprehensive imaging, artificial intelligence in classifying multimodal images has not always produced accurate results.

## 1. Introduction

Diabetic retinopathy (DR) has been one of the major causes of visual impairment and blindness. According to Sabanayagam et al., the annual incidence of DR ranges from 2.2% to 12.7%, and the progression ranges from 3.4% to 12.3% [1]. Moreover, a systematic review that examined the progression of DR to proliferative DR and severe vision loss in high-income countries showed a downward trend since the 1980s [2]. However, 80% of individuals with diabetes reside in developing countries, of which China and India comprise a large proportion [3]. Early diagnosis and prompt treatment of DR have been shown to prevent blindness [4]. While diabetic eye care has been mainly reliant on the number of ophthalmologists and necessary healthcare infrastructure [5], performing fundus examination, which is performed by ophthalmologists, for all patients with diabetes is unrealistic and expensive. Furthermore, expenses associated with DR have been substantial, whereas the financial impact may be even more severe given that several patients with this complication live in developing countries [6, 7], many of which have an inadequate number of ophthalmologists [8].

In contrast, automated image processing has proven to be a promising alternative for retinal fundus image analysis and its future application in eye care. Several recent studies have utilized state-of-the-art deep-learning (DL) algorithms for the automated detection of DR from a large number of fundus images [9–12]. In April 2018, the United States Food and Drug Administration approved the world's first artificial intelligence (AI) medical device for detecting DR, the IDx-DR. This AI system has allowed for specialty-level diagnostics to be applied in primary care settings [10, 13, 14], with studies expecting image diagnosis using AI to be a solution to the shortage of physicians and high medical expenses for specialists [15].

Several studies that examined the efficacy of automated detection have used standard fundus cameras that provide 30° or 50° images. In recent years, however, various fundus cameras have been developed, such as the ultra-wide-field (UWF) imaging fundus camera and optical coherence tomography angiography (OCTA).

UWF, otherwise known as Optos (Optos 200Tx; Optos Plc, Dunfermline, United Kingdom), is a non-contact, noninvasive imaging modality that can capture up to 200° of visible fundus and has become essential for understanding and managing the peripheral retinal pathologies of adult diseases such as diabetes and retinal vein occlusions [16, 17]. Indeed, one report showed the accuracy of UWF-based AI in the detection of DR [18].

OCTA has been devised to noninvasively detect moving objects within the fundus, such as flowing red blood cells, as a flow signal and visualize it as a blood vessel [19, 20]. In a similar manner, studies have suggested the accuracy of OCTA-based AI for detecting DR [21, 22].

However, manual analysis of multiple fundus images for accurate screening in clinical practice requires a substantial effort from ophthalmologists. As such, the objective of the present study was to investigate the accuracy of AI using different composite images.

## 2. Methods

### 2.1. Dataset

The study was approved by the Ethics Committee of Tsukazaki Hospital (Himeji, Japan) (no. 171001) and Tokushima University Hospital (Tokushima, Japan) (no. 3079) and was conducted in accordance with the tenets of the Declaration of Helsinki. Informed consent was obtained from either the participants or their legal guardians after the nature and possible consequences of the study (shown in Supplemental Human Studies Consent File 1) were explained to them.

The study dataset comprised 491 images and data from patients with diabetes. The data of those without fundus diseases between 2016 and 2019 were extracted from the clinical database of the ophthalmology departments of Saneikai Tsukazaki Hospital and Tokushima University Hospital. Images were reviewed by three retinal specialists to assess the presence of DR or NDR and registered in an analytical database. All patients underwent Optos (Optos 200Tx®, Nikon), OCTA (OCT Triton plus®, Topcon), and UWF fluorescein angiography. OCTA scans were acquired over a 6 × 6 mm^2^ region.

**Figure 1:**
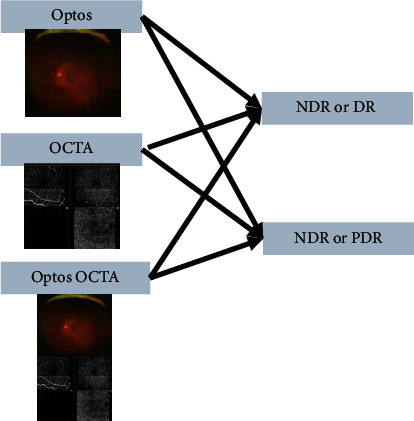
Identification of each image and stage. Test 1 (no apparent diabetic retinopathy [NDR] or diabetic retinopathy [DR]) and test 2 (NDR or proliferative diabetic retinopathy [PDR]) were performed using the Optos, optical coherence tomography angiography (OCTA), and Optos OCTA images.

En face images of the superficial plexus, deep plexus, outer retina, and choriocapillaris and the density map were extracted (Figure 1). DR levels were defined using the Early Treatment Diabetic Retinopathy (ETDRS) Severity Scale on the basis of the retinal images of the patients [4]. The 491 images that passed image-quality review were graded as follows: no apparent DR (NDR) (169 images), mild nonproliferative DR (NPDR) (76 images), moderate NPDR (54 images), severe NPDR (90 images), and proliferative DR (PDR) (102 images). All participants underwent comprehensive ophthalmological examinations, including slit-lamp biomicroscopy, dilated ophthalmoscopy, color fundus photography, and SS-OCTA. Data on age, sex, and previous hemoglobin A1c (National Glycohemoglobin Standardization Program) levels were obtained. Diabetes was diagnosed in accordance with the criteria of the 2016 Japanese Clinical Practice Guideline for Diabetes [23].

The present study examined the results of tests 1 and 2 to identify NDR and PDR. For each verification, Optos, OCTA, and Optos OCTA imaging were performed. We described how Optos OCTA images are created in the Image Processing Section.

This study used K-fold cross-validation (*k* = 5), which has been described in detail elsewhere [24, 25]. Briefly, image data were divided into K groups, after which *K* − 1 groups were used for training data, while one group was used for validation data. This process was repeated *K* times until each of the K groups became a validation dataset. The present study divided the data into nine groups. Images of the training dataset were augmented by adjusting for brightness, gamma correction, histogram equalization, noise addition, and inversion, which increased the amount of learning data 18 times. The deep convolutional neural network (DCNN) model was created and trained using data from preprocessed images, a method similar to those reported in previous studies [26, 27].

### 2.2. Image Processing

The aspect ratio of the original Optos images was 3900 × 3072 pixels. For analysis, the aspect ratio of all the images was changed and resized to 256 × 192 pixels.

**Figure 2:**
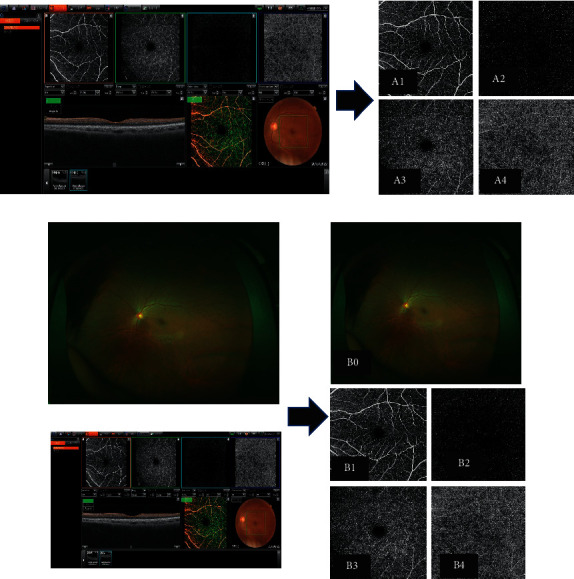
Test 1 (no apparent diabetic retinopathy [NDR] or diabetic retinopathy [DR]) and test 2 (NDR or proliferative diabetic retinopathy [PDR]) were performed using the Optos (a), optical coherence tomography angiography (OCTA), Optos (b), Optos OCTA images (A1–A4; B0–B4). A1, B1: Superficial OCTA image; A2, B2: deep OCTA image; A3, B3: other retinal layer of the OCTA image; A4, B4: choriocapillaris layer of the OCTA image; B0: Optos image.

The size of the concatenated original OCTA images was 640 × 320 pixels. The images of the four en face zones (superficial plexus, deep plexus, outer retina, and choriocapillaris) were extracted. The images of the superficial plexus, deep plexus, outer retina, and choriocapillaris were placed on the upper left, upper right, lower left, and lower right (Figure 2(a)), with the original input images resized to 256 × 192 pixels as the analysis time was reduced.

**Figure 3:**
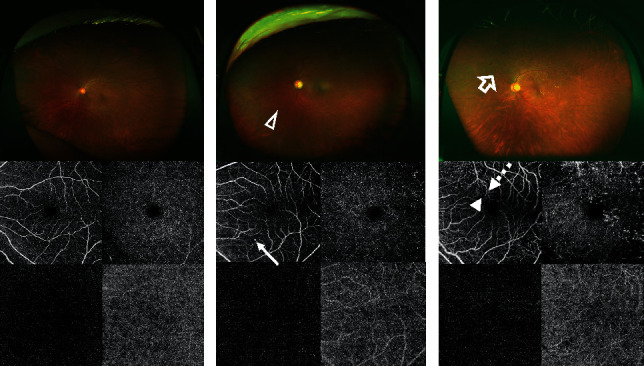
Representative images of no apparent diabetic retinopathy (a), mild nonproliferative diabetic retinopathy (b), and proliferative diabetic retinopathy (c) obtained using ultra-wide-field (UWF) imaging and optical coherence tomography angiography (OCTA). The UWF image shows the hemorrhage (white triangle) and neovascularization (white arrow). The OCTA image shows microaneurysm (white long arrow), microvascular tortuosity (white dotted arrow), and capillary non-perfusion (white short arrow).

The Optos OCTA image (Figure 2(b)) was created by combining Optos and the OCTA images vertically and resizing them to 256 × 192 pixels. Representative images of NDR, mild NPDR, and PDR are presented in Figure 3.

### 2.3. Deep Learning

**Figure 4:**
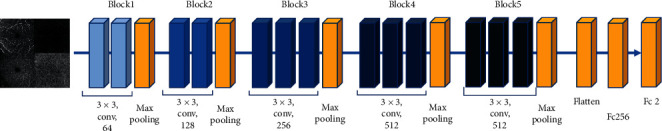
Visual geometry group (VGG) 16 model. The overall architecture of the VGG16 model is shown. The deep convolutional neural network used ImageNet parameters; the weights of blocks 1−4 and 5 were fixed, while the fully connected layers were adjusted.

In this study, a visual geometry group, −16 DCNN (VGG16) (Figure 4) [28], was used as the analytical model; the technical details of VGG16 will be described in the original paper, and the setup values for the present study will be described later. Before that, a brief outline for better understanding is given to the ophthalmologist.

### 2.4. Outline of VGG16

VGG16 automatically learns the local features of images and generates a classification model [29, 30]. It scans the entire image as often as 13 times in a small area (local receptive field) to see how many partial features (e.g., a long nose for an elephant and a long neck for a giraffe) the target image has. This scan is performed by moving the area pixel by pixel to examine the entire image comprehensively. It is called convolution because the resulting values are convolved into a single pixel value [29–31]. For example, if a whole image with 81 pixels (9 × 9 pixels) is scanned by shifting one pixel at a time in a local receptive field of 3 × 3 pixels, the scan is performed seven times in the horizontal direction and seven times in the vertical direction; thus, the scan result is compressed into 49 pixels (7 × 7). This means that the amount of information is collapsed to 60% (49/81). Furthermore, this feature is called a filter or channel. ReLU [32] was used as a function to highlight the feature extraction for each layer. An automatic adjustment called backpropagation is performed to strengthen or weaken the features to increase accuracy during the learning process of correct and incorrect answers. In VGG16, this feature pattern is increased from 64 types (called channels) to 128, 256, and 512 types as each block of the convolution process progresses. In addition, VGG16 also performs a process called max pooling five times, which reduces the number of pixels in each block of the convolution process by half for emphasizing features across the entire image (e.g., red tones for a fire scene and bright tones for a daytime photograph) [33]. The final combined layer (fully connected layer) accepts all the information from the previous layer without thinning it out and is responsible for linking it to probability values by passing through the Softmax function for binary classification, which is the purpose of this study.

### 2.5. VGG16 Settings Used in this Study

The aspect ratio of the original Optos images was 3900 × 3072 pixels, whereas that of the OCTA images was 640 × 320 pixels. For analysis, we changed the aspect ratio of all the input images and resized them to 256 × 192 pixels. Given that the RGB image input ranged from 0 to 255, we normalized it to a range of 0−1 by dividing it by 255. To increase the learning speed and improve performance even with a small amount of data, the initial weight values of the first four convolution blocks were used as parameters learned by ImageNet using the transfer learning method [34]. The Momentum Stochastic gradient descent algorithm was used to update the parameters of the model (learning ratio = 0.0005, inertial term = 0.9) [35, 36]. The construction and verification of the neural network were performed using a Python Keras (https://keras.io/ja/) with the backend as the tensorflow.

### 2.6. Outcome

This study evaluated the performance of six verifications, namely, tests 1 and 2 for Optos, OCTA, and Optos OCTA images. Receiver-operating characteristic (ROC) curves were created on the basis of the abilities of the DL models to discriminate between NDR and DR images (test 1), and between NDR and PDR images (test 2). These curves were evaluated using the area under the curve (AUC), sensitivity, and specificity. Sensitivity and specificity were considered positive (DR in test 1 and PDR in test 2) when the probability of the neural network output was greater than 0.5. The ROC curve was derived using Python scikit-learn (http://scikit-learn.org/stable/tutorial/index.html).

### 2.7. Statistical Analysis

To compare patient background, age was analyzed using Student's *t*-test, while the male-female ratios were compared using Fisher's exact test. In all cases, a *P* value of <0.05 was considered significant. All statistical processes were performed using Python Scipy (https://www.scipy.org/) and Python Statsmodels (http://www.statsmodels.org/stable/index.html).

For the AUC, the 95% confidential intervals (CIs) were obtained using the following formula [37]:

(1) $$\begin{array}{c} 95\%\text{CI}=A\pm 1.96\text{SE}\left(A\right). \end{array}$$

The mean AUC and SE(*A*) are the standard error of the AUC.

SE(*A*) was also obtained using the following formula [37]:

(2) $$\begin{array}{c} \text{SE}\left(A\right)=\sqrt{\frac{A\left(1-A\right)+\left(Np-1\right)\left(Q1-A^{2}\right)+\left(Nn-1\right)\left(Q2-A^{2}\right)}{Np\cdot Nn},} \end{array}$$

where *Np* is the number of blepharoptosis images, *Nn* is the number of normal images, *Q*1 is the probability that two randomly chosen abnormal images both ranked with greater suspicion than a randomly chosen normal image, and *Q*2 is the probability that one randomly chosen abnormal image ranked with a greater suspicion than two randomly chosen normal images.

*Q*1 and *Q*2 were obtained using the following formula:

(3) $$\begin{array}{c} Q1=\frac{A}{2-A},\\ Q2=\frac{2A^{2}}{1+A}. \end{array}$$

For sensitivity and specificity, 95% CIs were obtained using the Clopper-Pearson method [38].

(4) $$\begin{array}{c} \text{Clopper}-\text{pearson CI}\left(k,n\right)=\frac{k}{\left(n-k+1\right)F_{0.025}\left(2\left(n-k+1\right),2k\right)+k}∼\frac{\left(k+1\right)F_{0.025}\left(2\left(k+1\right),2\left(n-k\right)\right)}{\left(k+1\right)F_{0.025}\left(2\left(k+1\right),2\left(n-k\right)\right)+n-k}, \end{array}$$

where F_0.025_(*a, b*) is the 0.025 quantile from an F-distribution with a, *b* degrees of freedom, *k* is the number of successes, and *n* is the number of trials.

## 3. Results

### 3.1. Background

The baseline characteristics of the development and clinical validation datasets are described in Table 1.

### 3.2. Evaluation of Model Performance

In test 1, Optos, OCTA, and Optos OCTA images had an AUC of 0.790 (95% CI: 0.751–0.830), 0.883 (95% CI: 0.854–0.912), and 0.847 (95% CI: 0.814–0.880), respectively.

**Figure 5:**
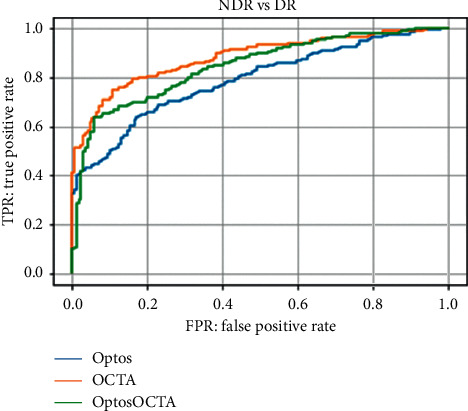
Receiver-operating characteristic curve for test 1 (no apparent diabetic retinopathy [NDR] or diabetic retinopathy [DR]) for Optos, optical coherence tomography angiography (OCTA), and Optos OCTA images. The order of imaging methods used based on the accuracy of their results was as follows: OCTA, Optos OCTA, and Optos.

The ROC curves are shown in Figure 5.

**Figure 6:**
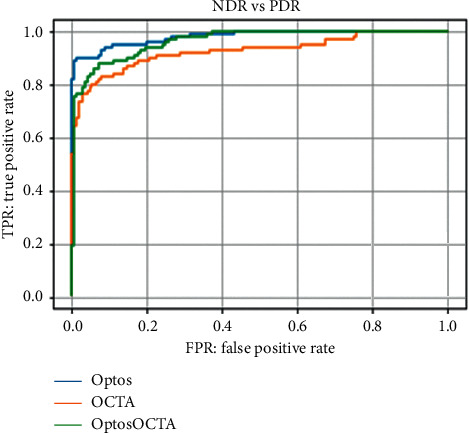
Receiver-operating characteristic curve for test 2 (no apparent diabetic retinopathy [NDR] vs. proliferative diabetic retinopathy [PDR]) for Optos, optical coherence tomography angiography (OCTA), and Optos OCTA images. The order of imaging methods used based on the accuracy of their results was as follows: Optos, Optos OCTA, and OCTA.

In test 2, the Optos, OCTA, and Optos OCTA images had AUC of 0.981 (95% CI: 0.962–1.064), 0.928 (95% CI: 0.892–0.964), and 0.964 (95% CI: 0.938–0.990), respectively. The ROC curves are shown in Figure 6.  Table 2 shows the sensitivity and specificity of the results of the analyses.

## 4. Discussion

The present study investigated the efficacy of the DL method in identifying the difference between NDR and DR on the basis of 491 multimodal images. The better DL algorithm showed appropriate sensitivity and specificity (AUC: 0.847; sensitivity: 78.6%; specificity: 69.8%), as well as good results with respect to differentiating NDR from PDR (AUC: 0.964; sensitivity: 80.4%; specificity: 96.4%). The ability to discriminate between NDR and PDR presented herein was comparable with that reported in previous studies [9–15]. All images in this study were obtained from patients with diabetes. Even patients with NDR showed significantly lower blood vessel density than healthy individuals, especially in the deep layer [39]. The multimodal imaging modality used in this study did not provide accurate results. Moreover, the multimodal images captured using AI were used in both tests 1 and 2, with the discriminative ability of Optos and OCTA being reversed in test 2.

First, OCTA with DL properly detected the difference between NDR and DR (test 1). The current international classification recommends diagnosis based on the presence of superficial retinal lesions. Therefore, the accuracy of OCTA, whose imaging range is narrower than that of UWF imaging, in determining the DR stage has generally been poor. However, OCTA images showed significant differences between NDR and DR even with an unevenly enlarged acicularity index and foveal avascular zone, indicating a relatively satisfactory staging accuracy [40]. When comparing patients with early-stage DR, imaging methods that show the local area are better than those that only show the whole area. Given that DR-related microvasculature damage may actually begin around the macula, narrow images can be expected to have the best predictive sensitivity for DR [41].

Second, Optos showed more accurate results in distinguishing NDR from PDR (test 2). Once a patient has developed DR, especially severe cases (e.g., PDR), a wider range of images can increase the diagnosis rate. Retinopathy lesions in DR that predominantly develop around the standard field defined in ETDRS 7 [42] are considered predominantly peripheral lesions, the extent of which is associated with retinopathy progression [43, 44]. Furthermore, this cohort included eyes treated with and eyes treated without a pan retinal photocoagulation (PRP) laser.

Progress in traditional technologies, such as digital fundus photography, along with recent advancements in various imaging modalities, has provided clinicians with new information and improved efficiency. Tran and Pakzad-Vaezi reported the benefits of multimodal imaging of DR and the clinical applications of several imaging techniques in DR including color photography, OCT, OCTA, and adaptive optics [45].

Furthermore, the use of the combination of DCNN and these multimodal images in diagnosing DR is expected to increase in the future, and the use of DCNN in the analysis of retinal images is appealing given its suitability with the current trend of teleophthalmology and telemedicine [46], and cost-effectiveness [47]. Considering that an automated DR grading software can potentially offer better efficiency, reproducibility, and early detection of DR, the use of this grading software in the screening of the even-increasing number of individuals with diabetes should help reduce the healthcare burden. The use of multimodal images with DCNN would enable screening for referable DR in remote areas where services of an ophthalmologist are unavailable. However, understanding the indications and limitations of each technology allows clinicians to gain the most information from each modality and thereby optimize patient care. In an actual human clinical setting, the combination of multiple imaging techniques can overcome their individual weaknesses and provide a more comprehensive representation. Such an approach helps in the accurate localization of a lesion and understanding the pathology in posterior segment. Considering that the major technological advancements in imaging over the past decade have improved our understanding and knowledge regarding DR, a multimodal approach to imaging has become the standard of care [48]. However, the present study revealed that multimodal diagnosis using AI did not always yield the best results.

The present study has several limitations. One of the major issues of this study is the small number of images for training. Many DL researchers agree that such a small number of data in each category is insufficient to test the effectiveness of the proposed method. Deep learning generally requires more than a million samples to train without overfitting. Another limitation is that this cohort included eyes treated with and eyes treated without a PRP laser, which may have confounded our results.

In summary, our study suggests that the use of AI in classifying multimodal images did not always produce accurate results and showed advantages and disadvantages depending on the stage. Although combination of DCNN and multimodal images certainly provides better result, it is not particularly superior to medical examination. Face-to-face examinations by ophthalmologists are indispensable for a definite diagnosis.

## 5. Conclusions

Although UWF fundus ophthalmoscopy and OCTA images with a DCNN were effective in diagnosing DR, the use of AI in diagnosing multimodal images did not always produce accurate results.

## Figures and Tables

**Table 1:** Patients' demographics.

	NDR	Mild	Moderate	Severe	PDR
Number of images	169	76	54	90	102
Patients	95	52	40	58	71
Women (%)	(42.6)	(40.8)	(38.9)	(35.6)	(34.3)
Mean age, years (SD)	66.8 ± 9.6	67.2 ± 9.7	67.4 ± 10.3	66.8 ± 8.6	59.0 ± 11.6
Left fundus (%)	(49.1)	(47.4)	(50.0)	(48.9)	(52.0)

NDR, no apparent diabetic retinopathy; PDR, proliferative diabetic retinopathy.

**Table 2:** Sensitivity and specificity values and 95% confidence intervals.

Test	Device	Sensitivity	Specificity
Test 1	Optos	80.9 (76.2–85.1)	55.0 (47.2–62.7)
	OCTA	83.9 (79.4–87.7)	71.6 (64.2–78.3)
	Optos OCTA	78.6 (73.7–82.9)	69.8 (62.3–76.6)

Test 2	Optos	90.2 (82.7–95.2)	97.0 (93.2–99.0)
	OCTA	74.5 (64.9–82.6)	97.0 (93.2–99.0)
	Optos OCTA	80.4 (71.4–87.6)	96.4 (92.4–98.7)

## Data Availability

The data that support the findings of this study are available from the corresponding author, Hitoshi Tabuchi, upon reasonable request.

## Supplementary Materials

Supplementary Materials The authors show the research consent form from the patient in the Supplemental Human Studies Consent File 1.
