# Supplementary material for: Accuracy of Diabetic Retinopathy Staging with a Deep Convolutional Neural Network Using Ultra-Wide-Field Fundus Ophthalmoscopy and Optical Coherence Tomography Angiography
Source: J Ophthalmol. 2021 Apr 3;2021:6651175. doi: 10.1155/2021/6651175 (PMC8041547; doi:10.1155/2021/6651175)
Supplement: Supplementary Materials — The authors show the research consent form from the patient in the Supplemental Human Studies Consent File 1. [file 6651175.f1.pdf]

Explanation and consent for the use of your treatment and test results in clinical research and clinical databases

To our patients
We are committed to providing the best possible medical care to our patients, and we are also committed to researching better ways to treat them. Even with the same treatment for the same disease, the treatment's effect may differ depending on each patient's background factors, or it may be unclear which of the multiple treatments is more effective.
In some cases, it is not clear which of the multiple treatments is more effective. Case studies, clinical research, multicenter collaborative research, clinical databases held by each department's academic society, and published in academic and medical journals may use your examination results to clear unresolved clinical issues.
Using your treatment details, test results, and stored specimens for research purposes, we promise that we never disclose your identifiable personal information to any third party outside the hospital.

The treatment or examination referred to in this consent form is a medical treatment or examination performed on a patient with the same disease as yours and approved by insurance. When we perform special tests or treatments to participate in clinical trials to confirm your treatment's effectiveness, we will provide you with sufficient explanation and obtain your consent.

Tsukazaki Hospital
Day ____________________

I agree with the above statements.

Address ____
Signature ____________________________________
